# Artificial intelligence with feature fusion empowered enhanced brain stroke detection and classification for disabled persons using biomedical images

**DOI:** 10.1038/s41598-025-14471-5

**Published:** 2025-08-09

**Authors:** Mohammed Alsieni, Khaled H. Alyoubi

**Affiliations:** 1 Department of Clinical Pharmacology, Faculty of Medicine, King Abdulaziz University, Jeddah, 21589 Saudi Arabia; 2 King Salman Centre for Disability Research, Riyadh, 11614 Saudi Arabia; 3 Faculty of Computing and Information Technology, King Abdulaziz University, Jeddah, Saudi Arabia

**Keywords:** Brain stroke, Biomedical images, Artificial intelligence, Disabled persons, Feature fusion, Transfer learning, Computational biology and bioinformatics, Mathematics and computing

## Abstract

Brain stroke is an illness which affects almost every age group, particularly people over 65. There are two significant kinds of strokes: ischemic and hemorrhagic strokes. Blockage of brain vessels causes an ischemic stroke, while cracks in blood vessels in or around the brain cause a hemorrhagic stroke. In the prompt analysis of brain stroke, patients can live an easier life. Recognizing strokes using medical imaging is crucial for early diagnosis and treatment planning. Conversely, access to innovative imaging methods is restricted, particularly in emerging states, so it is challenging to analyze brain stroke cases of disabled people appropriately. Hence, the development of more accurate, faster, and more reliable diagnostic models for the timely recognition and efficient treatment of ischemic stroke is greatly needed. Artificial intelligence technologies, primarily deep learning (DL), have been widely employed in medical imaging, utilizing automated detection methods. This paper presents an Enhanced Brain Stroke Detection and Classification using Artificial Intelligence with Feature Fusion Technologies (EBSDC-AIFFT) model. This paper aims to develop an enhanced brain stroke detection system for individuals with disabilities, utilizing biomedical images to improve diagnostic accuracy. Initially, the image pre-processing stage involves various steps, including resizing, normalization, data augmentation, and data splitting, to enhance image quality. In addition, the EBSDC-AIFFT model combines the Inception-ResNet-v2 model, the convolutional block attention module-ResNet18 method, and the multi-axis vision transformer technique for feature extraction. Finally, the variational autoencoder (VAE) model is implemented for the classification process. The performance validation of the EBSDC-AIFFT technique is performed under the brain stroke CT image dataset. The comparison study of the EBSDC-AIFFT technique demonstrated a superior accuracy value of 99.09% over existing models.

## Introduction

Currently, stroke is a significant medical issue. A cerebrovascular event, commonly known as a stroke, is a neurological disorder that can occur because of an obstruction or bleeding in the brain’s blood vessels, often causing varied physical and mental impairments that affect daily functioning. Stroke is a significant reason for long-term disability in many developed nations^1^. Every year, stroke affects around 16 million people globally. Stroke is a diverse set of conditions marked by sudden, localized interruption of blood flow to the brain, causing neurological symptoms lasting more than 24 h^2^. Ischaemic strokes arise whenever blood vessels are blocked by an embolus or thrombus, leading to brain ischemia. Hemorrhagic strokes occur due to the break and bleeding of a damaged blood vessel into brain tissues, which typically results in increased intracranial pressure^3^. The first one is connected to medical conditions, like diabetes mellitus, cardiac diseases, and lifestyle factors like poor nutrition, obesity, and consumption of alcohol and tobacco. On the other hand, the second one is of gender, age, ethnicity, and race^4^. A stroke may result in loss of function as it damages brain tissue responsible for various functions, including sensation, movement, cognition, and speech. This injury results in impairment, affecting daily activities^5^. Hence, stroke-related disability is physical, cognitive, or communicative. Initial detection and improved management of modifiable risk factors are the basis for preventing stroke and avoiding the later evolution of vascular dementia^6^. Brain Stroke classification for disability people contains a complete medical background, a neurologicalas well as physical test, and a brain imaging examination (for instance, magnetic resonance imaging (MRI) or computed tomography (CT) scans) to eliminate other stroke imitators and to identify the type of stroke, its location and damage level^7^. Stroke treatment emphasizes two key objectives: reducing brain damage and preventing further complications. The particular treatment method relied on the kind of stroke (ischemic or hemorrhagic) and post-stroke time.

Currently, DL methods have developed and advanced rapidly in various domains, including healthcare systems^8^. It classifies ischaemic heart diseases and strokes as the dual primary reasons for death and disability around the world. Hospital expenses for stroke are rising, and therefore, there is an urgency for sophisticated techniques that aid in medical diagnosis, treatment, clinical action estimations, and recommendations for potential treatment approaches, as well as rehabilitation programs^9^. Initial identification of stroke is a vital stage for effective treatment, and DL is extremely useful in this procedure. DL is a crucial technology that assists medical personnel in making clinical judgments and predictions^10^. At present, many studies are performed on the enhancement of brain stroke identification for disabled individuals utilizing DL concerning precision and rapidity.

This paper presents an Enhanced Brain Stroke Detection and Classification using Artificial Intelligence with Feature Fusion Technologies (EBSDC-AIFFT) model. This paper aims to develop an enhanced brain stroke detection system for individuals with disabilities, utilizing biomedical images to improve diagnostic accuracy. Initially, the image pre-processing stage involves various steps, including resizing, normalization, data augmentation, and data splitting, to enhance image quality. In addition, the EBSDC-AIFFT model combines the Inception-ResNet-v2 model, the convolutional block attention module-ResNet18 method, and the multi-axis vision transformer technique for feature extraction. Finally, the variational autoencoder (VAE) model is implemented for the classification process. The efficacy of the EBSDC-AIFFT technique is tested under the brain stroke CT image dataset. The key contribution of the EBSDC-AIFFT technique is listed below.

- The EBSDC-AIFFT model utilized a comprehensive image pre-processing pipeline that included resizing, normalization, data augmentation, and data splitting to improve image quality and facilitate effective model training. These steps enhanced the diversity and representativeness of the dataset, thereby mitigating overfitting and improving the generalizing capability. As a result, the model achieved more reliable and accurate predictions across a variety of input conditions.
- The EBSDC-AIFFT approach employs a hybrid feature extraction framework that integrates Inception-ResNet-v2, CBAM-ResNet18, and MaxViT techniques for capturing a wide range of complementary image features. This fusion model improves the capability to represent complex patterns and improves robustness across different image types. The integrated approach yields more comprehensive and discriminative feature representations, thereby enhancing overall classification accuracy.
- The EBSDC-AIFFT methodology employs a VAE-based classification approach to enhance model robustness and accuracy by leveraging probabilistic latent representations. This method enables the capture of complex data distributions, improving generalization on unseen data. By utilizing these latent features, the model achieves more reliable and precise classification results.
- The integration of advanced fusion-based feature extraction with VAE-based classification presents a novel and effective framework that significantly improves feature representation and classification robustness. This unique integration leverages the complementary merits of multiple extraction techniques in conjunction with probabilistic latent modelling. As a result, it surpasses conventional DL methods by capturing richer data patterns and improving model generalization, presenting a distinctive approach.

## Related works

Bhandari et al.^11^ presented a web-based stroke risk evaluation device that distinctly integrates a robust ML model. This model employs a novel integration of logistic regression and SMOTE oversampling, refining the recognition of stroke risk features compared to existing approaches. Initial detection of people at serious risk of stroke can drastically enhance preventive care results. Gencer and Gencer^12^ analyzed DL-enabled methods for automated multi-class classification of brain strokes, and a novel method merging quantum genetic algorithms (QGA) and DL is presented. The robust feature extraction method, EfficientNetB0, was leveraged and combined with QGA, introducing a novel technique. It is intended to create a feature selection (FS) technique. Kanchanamala et al.^13^ proposed a new brain tumour identification method depending on the exp-walruses hunting optimizer-based squeezeNet (EWHO-SqueezeNet) model. Next, the denoising and Region of interest (RoI) were carried out in the pre-processing stage. Then, M − SegNet is used for segmentation. Following that, feature extraction is performed. Finally, SqueezeNet is utilized to detect brain tumours, which is adjusted via the established exponential-walrus-hunting optimizer (EWHO) technique. Ye et al.^14^ introduced a new technique to enhance the configuration of artificial and convolutional neural network (ANN) and (CNN) methods for solving the BTD problem. This method utilizes CNN to segment brain MRIs. The genetic algorithm (GA) and multi-linear principal component analysis (MPCA) are employed for tuning and reducing the segmented feature dimensions. Pacal et al.^15^ presented a unique adaptation of the EfficientNet-v2 framework, enhanced with efficient channel attention (ECA) and a global attention mechanism (GAM), which addresses these difficulties. Yang and Razmjooy^16^ proposed a new ML approach, which merges the gains of the enhanced hybrid dwarf mongoose optimizer (EHDMO) method and gated recurrent unit (GRU) model to detect brain tumours. To utilize the EHDMO method for fine-tuning the GRU network’s parameters. Rajendran et al.^17^ employed a grey-level co-occurrence matrix feature extraction model to remove irrelevant facts from the images. In contrast to the present advanced methods, the precision of brain tumour classification was considerably improved by CNNs. By integrating the outcomes of a dual, distinct segmentation network, this approach exhibits a vital but modest combinatorial approach that, as a direct result, generates more accurate and broad estimations. Poonguzhali et al.^18^ suggested an automatic deep residual UNet segmentation with a classification method (ADRU-SCM) for diagnosing brain cancers. This model primarily aims to segment and classify BT. Moreover, this model employs a deep residual U Net segmentation approach. Additionally, the VGG19 technique was utilized as a feature extraction method.

Khalafi et al.^19^ proposed a model by using approaches such as Xception, EfficientNet, Inception, ResNet, VGG, and ML models like random forest (RF) and support vector machine (SVM). The approach aims to enhance non-invasive, cost-effective diagnosis and prognostic evaluation of strokes. Abulfaraj et al.^20^ proposed an advanced brain stroke (BS) detection framework that integrates MobileNet V3 for deep feature extraction from MRI images, integrated with ensemble learning (EL) using light gradient boosting machine (LightGBM), categorical boosting (CatBoost), and RF classifiers. Das et al.^21^ developed an automated rehabilitation evaluation system for stroke patients using a hybrid approach that integrates a Fuzzy Logic Rule-Based System (FLRBS) and a K-Nearest Neighbour (K-NN) approach. The FLRBS evaluates joint angles and range of motion during exercises, while K-NN classifies exercise accuracy, enabling real-time feedback and supporting telerehabilitation. Akolgo et al.^22^ explored the transformative potential of AI in healthcare by analyzing its applications. Wang et al.^23^ improved early diagnosis and personalized treatment of acute ischemic stroke (AIS) by integrating radiomics, machine learning (ML), and DL techniques. The approach leverages radiomic feature extraction and multimodal neuroimaging data fusion to improve prediction accuracy and clinical decision-making for AIS management. Tang et al.^24^ presented a comprehensive smart home rehabilitation system for post-stroke patients by utilizing a combination of IoT architecture, ML, and a large language model (LLM)-based agent. The platform integrates plantar pressure analysis, eye-tracking for cognitive monitoring, and ambient sensors to deliver real-time, adaptive, and privacy-aware rehabilitation support in home settings. Occhipinti et al.^25^ proposed an integrated smart home rehabilitation system for post-stroke care using wearable sensors, ambient monitoring, and an LLM-powered agent. The model utilizes ML-based plantar pressure arrays, a wearable eye-tracking module for cognitive monitoring, and smart home automation enhanced by LLM-based real-time assistance (Auto-Care), presenting continuous, personalized support with high accuracy and user satisfaction. Dhinakaran et al.^26^ presented an advanced multi-disease prediction framework utilizing electronic health records (EHR) by integrating the stabilized energy valley optimization with enhanced bounds (SEV-EB) model. Additionally, a hybrid short-term contextual attention network (HSC-AttentionNet) technique integrating temporal convolution and LSTM is employed to improve predictive accuracy and capture complex temporal dependencies in health data. Goswami et al.^27^ investigated the integration of passive brain-computer interface (BCI) technologies with ambient assisted living (AAL) systems, utilizing AI for advanced signal processing, cognitive pattern recognition, and adaptive environment control.

Cai et al.^28^ proposed the M3 Stroke tool, which utilizes a MultiModal Mobile AI framework integrating audio-visual data and edge-based DL methods to improve early triage of ischemic stroke, particularly in patients with mild or moderate symptoms. Utilizing DL and mobile computing, the system presents real-time, accurate stroke classification and supports telemedicine applications on iOS devices. Kina^29^ developed a rapid and efficient method by a lightweight convolutional architecture based on EfficientNet integrated with a squeeze attention block and transfer learning (TL). The model also incorporates the synthetic minority oversampling technique (SMOTE) to address data imbalance and uses gradient-weighted class activation mapping (Grad-CAM) for explainability. Qasim et al.^30^ presented a technique by utilizing a deep neural network (DNN) method with a weighted binary cross entropy (BCE) loss function. By analyzing factors such as age, gender, hypertension, and lifestyle variables, the model enhances the detection of critical stroke cases. Inamdar et al.^31^ proposed a novel dual-stream DL technique by utilizing a hybrid dual attention mechanism (DAM), multi-scale feature extraction module, and adaptive random vector functional link (ARVFL) approach to accurately classify ischemic stroke from computed tomography (CT) images. Qasrawi et al.^32^ presented an approach by utilizing a hybrid model that integrates stroke precision enhancement model (SPEM), ensemble DL, and intelligent lesion detection and segmentation techniques. Nivodhini et al.^33^ developed a GA-optimized bidirectional long short-term memory (BiLSTM) network framework that effectively captures temporal dependencies in multimodal neuroimaging data for accurate stroke diagnosis. The integration of GA improves model parameter selection to enhance diagnostic sensitivity and specificity across diverse clinical settings. Mena et al.^34^ proposed a technique by employing CNN-based lesion segmentation with the CLCI-Net model with both DL and shallow ML classifiers for vascular territory localization in stroke-affected MRI images. By integrating advanced image preprocessing techniques, the model achieves high accuracy in stroke lesion detection and classification. Gnanabaskaran et al.^35^ developed an intelligent framework for early cerebral stroke diagnosis by incorporating a pre-trained Visual Geometry Group 16 (VGG16) model with support vector machines (SVM) for precise classification. This integration utilizes DL and conventional ML models to improve diagnostic accuracy using medical imaging data. Wang et al.^36^ introduced an automated classification system for cognitive and motor impairments in stroke patients using 3D brain MRI. It utilizes radiomics and fusion feature extraction, followed by classification using 14 ML models comprising RF and linear discriminant analysis (LDA), along with model interpretation using SHapley Additive exPlanations (SHAP) to support clinical decision-making. Comparison analysis of existing brain strokes for disabled persons in Table 1.

**Table 1:** A comparative study of existing studies on brain stroke detection and classification.

Author	Year	Objective	Method	Dataset	Result
Bhandari et al.^11^	2025	To project a web-based stroke risk evaluation device that exclusively integrates a user-friendly interface with strong ML techniques.	Logistic Regression (LR) And SMOTE.	Stroke Prediction Dataset	Accuracy of 93.2%.
Gencer and Gencer^12^	2025	To enhance the effectiveness of AI and DL techniques in BT categorization, facilitating quicker and more accurate prior identification.	CNN, EfficientNetB0, and Quantum GA (QGA)	Figshare Brain Tumor and MRI Brain Tumor Datasets.	Higher accuracy of 98.36% and 98.25%.
Kanchanamala et al.^13^	2025	Early BT classification aids the former analysis of BTs by allowing the recognition of abnormal brain tissue.	SqueezeNet, Exponential-Walruses Hunting Optimization, Exponential Deer Hunting Optimization, and M − SegNet	-	High accuracy, True Positive Rate, and True Negative Rate of 93.20%, 95.18%, and 93.78%.
Ye et al.^14^	2024	To meet the requirement of enhancing the configuration of neural learning techniques by the features of the BTD concern.	CNN, Multi-Linear Principal Component Analysis (MPCA), ANNs, and GA.	BRATS2014 and BTD20 Datasets.	Accuracy of 98.6% and 99.1%.
Pacal et al.^15^	2024	A novel CNN framework enhanced with an attention mechanism, projecting a vital alternative that outperforms both VT and classical CNN.	EfficientNetv2	Brain Tumor MRI Dataset.	Accuracy of 99.76%.
Yang and Razmjooy^16^	2024	A novel approach to identify the BTs.	GRU and EHDMO.	Brain-Tumor-Progression Dataset.	Sensitivity of 0.98, specificity of 0.97, PPV of 0.98, NPV of 0.98, and accuracy of 0.95.
Rajendran et al.^17^	2023	Segmenting automatically utilizing MR data is vital for disease examination and monitoring.	Grey Level Co-OCCURANCE Matrix (GLCM), Vantage Point Tree (VPT), and U-Net.	MRI Dataset.	F-Score is 99.40% and Sensitivity is 99.39% Sensitivity.
Poonguzhali et al.^18^	2023	A primary tumour brain identification recommends an earlier response to treatment, which aims to increase the survival rate of patients.	Weiner Filter (WF), Deep Residual U-Net, VGG19, GRU, And Tunicate Swarm Optimization (TSO).	FigShare Dataset.	Accuracy is 97.48%.
Khalafi et al.^19^	2025	To improve stroke detection.	Xception, EfficientNet, Inception, ResNet, VGG, RF, SVM.	Retinal Imaging Data Linked to Stroke Diagnosis	Up to 98% Accurate.
Abulfaraj, Dutta, and Sait^20^	2024	To develop an accurate BS detection model to assist in early diagnosis through MRI image analysis.	MobileNet V3, LightGBM, CatBoost, RF.	Public Clinical MRI Dataset with 2888 Images	Accuracy of 98.7%, AUC of 0.95.
Das et al.^21^	2025	To enhance stroke rehabilitation through the use of ML techniques.	FLRBS, K-NN, Real-Time Angle Tracking, Range of Motion Analysis.	Exercise Data from 30 Physical Therapists.	Up to 98% Accurate.
Akolgo et al.^22^	2024	To review the transformative role of AI across healthcare domains.	Comprehensive Literature Review, AI Application Synthesis, Ethical & Practical Analysis	Not Applicable (Literature-based Study)	N/A
Wang et al.^23^	2025	To improve early diagnosis and personalized treatment of AIS.	Radiomic Feature Extraction, Multimodal Data Fusion, ML and DL, Neuroimage-Based Prediction.	Neuroimaging Data from Multiple Clinical Studies	Up to 92% Accurate.
Tang et al.^24^	2024	To develop a smart home platform.	Plantar Pressure Sensing, Eye-Tracking Integration, Ambient IoT Monitoring, LLM	Post-Stroke Patient Data	Up to 94%
Occhipinti et al.^25^	2025	To develop a smart home rehabilitation platform integrating wearable and ambient sensors with LLM.	Plantar Pressure Classification, Wearable Eye-Tracking Analysis, Ambient Sensor Control, LLM-Powered Real-Time Agent.	Custom Sensor-based Data from Smart Home Trials	94% Classification Accuracy, 100% Control Success.
Dhinakaran et al.^26^	2024	To improve multi-disease prediction for temporal pattern modelling.	SEV-EB feature selection, Statistical + deep features, HSC-AttentionNet model, EL.	EHR Data	95% Accuracy, 94% F1-Score
Goswami et al.^27^	2025	To explore the integration of passive BCI technologies through advanced AI methods.	Passive BCI Signal Analysis, AI, Smart Environment Adaptation, Cognitive Signal Interpretation.	Neural Signal Datasets (Varied)	Not Explicitly Reported.
Cai et al.^28^	2024	To develop and deploy a multimodal AI-powered mobile tool for the accurate triage of mild or moderate ischemic stroke patients.	Audio-visual multimodal AI, Mobile edge computing, DL.	269 Patient Recordings (191 Stroke, 78 Non-Stroke)	80.85% Accuracy, 90.63% Sensitivity.
Kina^29^	2025	For rapid and effectual brain condition detection using MRI.	EfficientNet, Squeeze Attention Block, TL, SMOTE, Grad-CAM	Imbalanced MRI Datasets, Balanced Training Data (via SMOTE)	Model exhibits high efficiency and explainability.
Qasim et al.^30^	2024	To develop a computationally efficient model to improve stroke risk prediction.	Weighted BCE, DNN, Demographic & Health Data Analysis	Demographic and Health Records	Recall, Precision, F1-Score
Inamdar et al.^31^	2025	To develop a dual-stream DL framework.	DAM, Multi-Scale Feature Extraction, ARVFL	Single- and Multi-center CT Dataset	High Accuracy, Strong Generalization
Qasrawi et al.^32^	2024	To enhance the detection and classification of ischemic brain strokes using a hybrid model.	SPEM, Ensemble DL, Lesion Detection & Segmentation	10,000 CT-Scans	Improved Accuracy (up to 98.2%)
Nivodhini et al.^33^	2025	To develop a model for accurate and efficient stroke diagnosis using neuroimaging data.	GA, BiLSTM	CT and MRI Scans	High Sensitivity, Specificity
Mena et al.^34^	2024	To develop an end-to-end AI-based framework for accurate stroke lesion segmentation and classification.	Image Preprocessing, CLCI-Net, DL and Shallow ML	MRI Brain Images	84% Accuracy (DL), 95% Accuracy (ML)
Gnanabaskaran et al.^35^	2025	To develop an intelligent framework for accurate early diagnosis of cerebral strokes.	Pre-trained VGG16, Feature Extraction, SVM	Annotated Brain Scans	96.50% Accuracy
Wang et al.^36^	2024	To automatically classify motor and cognitive disorders in stroke patients using 3D brain MRI and ML models.	Radiomics and fusion Features, 14 ML Models, Ensemble Classification, SHAP-Based Model Explainability	3D OAx T2 Propeller MRI Scans	92.0% Cognitive Accuracy, 82.5% Motor Accuracy

Despite various improvements in stroke diagnosis and rehabilitation using DL, ML, and hybrid AI models, several limitations still exist. Several techniques depend on large, annotated datasets, which are not uniformly available across diverse clinical settings, restricting generalizability. Methods such as SMOTE and GA-based optimization address imbalance and parameter tuning but may introduce overfitting risks. Complex hybrid models such as EHDMO-GRU or SPEM-Ensemble DL are often computationally intensive and inappropriate for real-time deployment. Research gap exists in the integration of lightweight, explainable models with high interpretability while maintaining performance. Additionally, limited work addresses multi-modal neuroimaging fusion at scale using unified frameworks. Most existing approaches lack robust validation on multi-center, heterogeneous datasets. Also, integration with IoT/IoMT and AAL remains fragmented without consistent privacy-preserving or real-time adaptive capabilities.

## Proposed methods

**Fig. 1:**
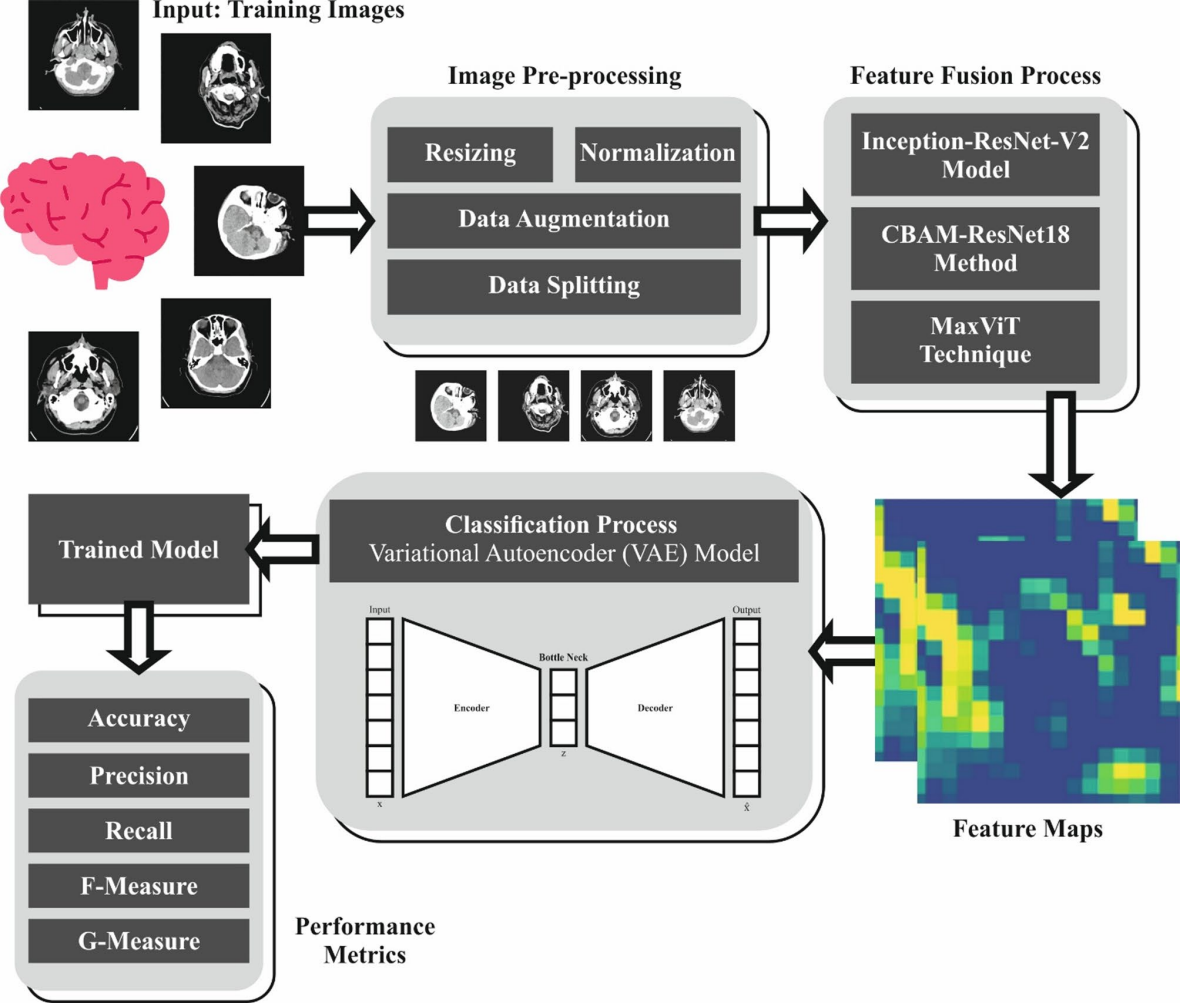
Workflow process of the EBSDC-AIFFT model.

This paper develops an EBSDC-AIFFT model. This paper aims to create an enhanced brain stroke detection system for individuals with disabilities, utilizing biomedical images to improve diagnostic accuracy. To achieve this, the EBSDC-AIFFT model comprises several stages, including image pre-processing, feature extraction, and classification. Figure 1 depicts the complete working flow procedure of the EBSDC-AIFFT approach.

### Image pre-processing

Initially, the image pre-processing phase involves multiple steps to enhance image quality. To safeguard compatibility and consistency with the method input requirement, the succeeding pre-processing stages are utilized^37^:

- Image Resizing: All images are resized to 256 × 256 using bilinear interpolation, ensuring consistency and preserving key stroke-related features.
- Normalization: Pixel intensities are scaled between 0 and 1 by dividing each pixel by 255. This scaling step enhances the training efficiency of the model by standardizing the input feature range for faster convergence.

1 $$\:{I}_{normalized}=\frac{{I}_{original}}{255}$$

- Data Splitting: Distinct epochs. It ensures that the method is trained on a substantial portion of the data, whereas distinct validation and testing sets provide an unbiased assessment of its performance.
- Data Augmentation: To improve the generalization capability of the methodology and reduce data augmentation and overfitting, models are trained on the training set. These methodologies comprise width, rotation, shear, height shifts, horizontal flipping and zoom. To present variability in training data, it is transformed into hidden data.
- Rotation: For an angle $\:\theta\:$, the rotation matrix was.

2 $$\:R\left(\theta\:\right)=\left[\begin{array}{cc}cos\left(\theta\:\right)&\:-sin\left(\theta\:\right)\\\:sin\left(\theta\:\right)&\:cos\left(\theta\:\right)\end{array}\right]$$

- Translation (Shifting): For a shift in $\:x$ and $\:y$ directions by $\:{\delta\:}_{x}$ and $\:{\delta\:}_{y}$:

3 $$\:T\left({\delta\:}_{x},{\delta\:}_{y}\right)=\begin{array}{ccc}1&\:0&\:{\delta\:}_{x}\\\:0&\:1&\:{\delta\:}_{y}\\\:0&\:0&\:1\end{array}$$

- Scaling: To measure an image by factor$\:s$, the scaling matrix was:

4 $$\:S\left(s\right)=\left[\begin{array}{cc}s&\:0\\\:0&\:s\end{array}\right]$$

### Fusion feature extraction techniques

Additionally, the EBSDC-AIFFT model combines the Inception-ResNet-v2 model, the CBAM-ResNet18 method, and the MaxViT technique for feature extraction. The fusion model is chosen to harness the complementary strengths of the two models in feature extraction. Inception-ResNet-v2 combines the efficiency of Inception modules with residual connections, allowing for deep and diverse feature learning while mitigating vanishing gradient issues. The CBAM-ResNet18 model is incorporated to enhance the capability to focus on relevant spatial and channel-wise features, thereby improving interpretability and performance. MaxViT introduces a novel attention mechanism that efficiently captures both local and global dependencies, thereby enhancing the technique’s capacity to comprehend complex patterns. The model achieves superior feature representation by integrating these techniques, striking a balance between accuracy and computational efficiency, and outperforms single-model approaches in terms of robustness and generalization across various datasets. This fusion also enables the model to capture better subtle and hierarchical features that are critical in complex classification tasks.

#### Inception-ResNet-v2 model

In the Inception structure, the use of 1 × 1, 3 × 3, and 5 × 5 convolutional kernels on dissimilar branches enables feature extraction at various spatial scales. Features extracted from these branches are then connected along with the channel size to provide robust feature representations^38^. This model permits for effective growth of either network width or depth, thus improving precision and alleviating overfitting. Additionally, the Inception structure offers the benefits of reducing parameter counts and enhancing network nonlinearity. The Residual block, as presented in ResNet, effectively addresses the problems of exploding and vanishing gradients in the training of DNNs. It presents a skip connection between network layers, allowing input to bypass intermediate layers and proceed directly to the subsequent layers. This model retains the novel information and alleviates the challenges associated with training deep techniques. The Inception-ResNet structure integrates the strengths of ResNet and Inception by utilizing the Inception framework to incorporate residual blocks, thereby further enhancing the network’s convergence speed. In this work, the InceptionResNet-v2 method is used. The framework comprises a Stem module, five Inception-ResNet-A modules, a Reduction-A module, ten Inception-ResNet-B modules, a Reduction-B module, and five Inception-ResNet-C modules. Additional layers include a Softmax, a pooling layer, and a dropout layer. The Stem module employs a parallel architecture and asymmetric convolutions to reduce complexity while preserving information. The following modules accelerate training, mitigate gradient vanishing, and eliminate either non-sparse or sparse features.

#### CBAM-ResNet18 model

ResNet is one of the neural networks projected to allow the training of DL techniques with various depths. This framework is designed to address common concerns in DL training, such as longer training periods and limited layer counts^39^. ResNet utilizes residual connections to pass data across layers, mitigating learning degradation and enabling efficient training of deep networks. The benefits of ResNet contrast with those of another framework, and its performance doesn’t diminish its deep learning capabilities. It comprises connections that pass over 2 to 3 layers, including batch normalization (BN) and ReLU among frameworks:

5 $$\:y=F\left({x}_{i}W+x\right)$$

$\:W$ depicts a weighted matrix, $\:x$ and $\:y$ refer to input and output layers, and the $\:F$ function indicates residue mapping. In a ResNet framework, a residual block is applied if the output and input dimensions of every block are similar. The layer counts generate every residue block that relies on ResNet. For the ResNet-8 and ResNet-34 methods, every residue block comprises dual layers. Simultaneously, for the deeper ResNet-50 and ResNet-10$\:1$ techniques, every residue block comprises three layers. ResNetCBAM comprises various major modules, including channel and spatial attention modules, that function to emphasize the significant features of the input data.

ResNetCBAM is a DL technique which integrates the ResNet framework with CBAM on the attention module. This method is intended to enhance the capability to identify relevant attributes of image data. In the ResNetCBAM model, the ResNet structure employed is ResNet 18, which comprises 18 convolutional layers. Furthermore, the convolution layer, ResNet-18, also uses a BN and max pooling layer to enhance the efficacy and performance of the methodology. CBAM includes dual central attention mechanisms (AMs): Spatial Attention and Tractal Attention. The channel assigns diverse attention to every channel in mapping features, allowing the methodology to concentrate on the most informative channel. Concurrently, each spatial attention position directs diverse attention to every spatial position in mapping features, permitting the method to focus on the most relevant area in an image. Within this ResNetCBAM technique, the CBAM module is effectually combined with the ResNet-18 structure. Once various layers of pooling and convolution in ResNet-18 have been applied, the mapping feature is fed to the CBAM module.

An improved feature mapping with superior weight of attention to appropriate regions and features. These dual AMs work together to enhance the methodology’s capability to acquire and utilize meaningful data from input image information. The feature extraction procedure initiates to retrieve the output from the ResNet 18 structure. Additionally, the mapping features are modified to incorporate the channel attention map (CAM) into the element-wise multiplication process. The outcome of this operation is modified mapping features, which aim at regions and features which is most relevant for specific tasks.

6 $$\:R=CAM\odot\:F$$

The mapping feature specified that $\:F$ is the output improved to utilize the CBAM attention mechanism. CAM highlights the significance of each channel in feature mapping, allowing the model to concentrate on key aspects. Operation $\:R$ multiplies the feature map with dual CAMs to refine representation. CBAM, integrating spatial and channel attention, assists ResNet-CBAM extract meaningful information effectively.

#### MaxViT technique

The MaxViT module includes an attention mechanism with blocks, grids, and MBConv^40^ MBConv feature maps are sent to grid and block attention modules, enhancing parameter efficiency and computational speed—ideal for edge or mobile devices. Block attention targets local feature aggregation, while grid attention improves global feature representation. MaxViT uses MBConv as its core convolution unit. The block of MBConv is expressed:

7 $$\:{X}_{MBCov}=X+proj\left(SE\left(DWCon{v}_{3\times\:3}\left(Con{v}_{1\times\:1}\left(Norm\left(X\right)\right)\right)\right)\right)$$

Here, $\:Norm$ represents the batch normalization, and $\:Con{v}_{1\times\:1}$ indicates the convolution process with a 1 × 1 kernel size. $\:X$ and $\:{X}_{MBConv}$ refer to the input and output mapping features. $\:SE$ denotes SE layer, $\:proj$ specifies a convolution operation to decrease channel counts, and $\:DWCon{\nu\:}_{3\times\:3}$ specifies depth-wise convolution with a 3 × 3 kernel size. In blocks of MaxViT, every attention operator employed is relative attention described in Eq. (8):

8 $${Rel}\, Atention\left( {Q,K,~V} \right) = softmax\left( {\frac{{QK^{T} }}{{\sqrt d }} + B} \right)y$$

Now $\:Q,K,\:V\in\:{R}^{(H\times\:W)\times\:C}$ refers to query, key, and value matrices, $\:\left(H\times\:W\right)\times\:C$ represents a dimension of these matrices, here $\:W$ and $\:H$ indicate the width and height of mapping features, correspondingly, $\:d$ depicts hidden dimension, $\:B$ denotes learned static location-aware matrix and $\:C$ represents channel counts. Within the block attention module, the hyper-parameter $\:P$ is described to split an input mapping feature $\:X\in\:{R}^{H\times\:W\times\:C}$ into $\:\frac{H}{P}$ non-overlapping size blocks $\:P\times\:P$.

9 $$\:\left(H,\:W,\:C\right)\to\:\left(\frac{H}{P}\times\:P,\frac{W}{P}\times\:P,\:C\right)\to\:\left(\frac{HW}{{P}^{2}}\times\:P,{P}^{2},\:C\right)$$

Subsequently, the relative attention to the 2nd dimension is carried out on the local characteristic. The forward of grid attention is defined in Eq. (10):

10 $$\:{X}_{Block}={X}_{MBConv}+UnBlock\left(RelAtention\left(Block\left(LN\left({X}_{MBCon}\right)\right)\right)\right)$$

Here, $\:UnBlock\:\left(\right)\:$and $\:Block\:(\cdot\:)$ refer to reverse- and block partition$\:.\:LN$ signifies layer normalization. Likewise, the mapping features are separated by $\:G$ lattices of size $\:\frac{H}{G}\times\:\frac{W}{G}$ by hyperparameter $\:G$, and the shape of mapping features is defined in Eq. (11):

11 $$\:\left(H,\:W,\:C\right)\to\:\left(G\times\:\frac{H}{G},\:G\times\:\frac{W}{G},\:C\right)\to\:\left({G}^{2},\frac{HW}{{G}^{2}},\:C\right)$$

12 $$\:{X}_{Grid}={X}_{Block}+UnGrid\left(RelAttention\left(Grid\:\left(LN\left({X}_{Block}\right)\right)\right)\right)$$

**Fig. 2:**
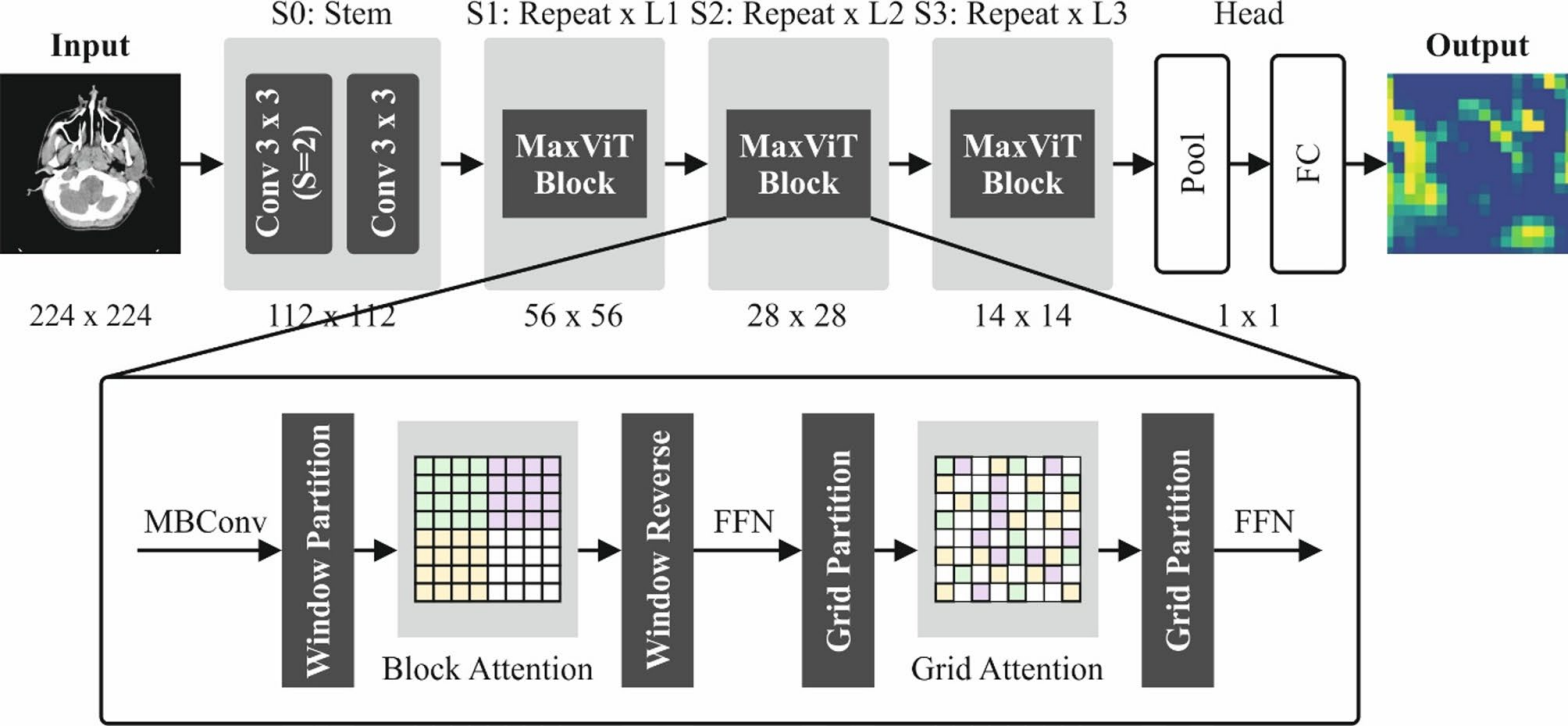
Structure of the MaxViT model.

Where $\:Grid\:(.)\:$and $\:UnGrid(.)$ refer to grid and reverse grid partitions, respectively. Figure 2 represents the MaxViT model.

### VAE-based classification model

At last, the VAE model is employed for the classification process^41^. This model is chosen for its robustness in learning meaningful latent representations from complex and high-dimensional data. Unlike conventional autoencoders, VAEs impose a probabilistic framework that encourages smooth and continuous latent spaces, thereby enhancing generalization and robustness to noise. The model demonstrates efficiency in capturing subtle data discrepancies, particularly improving classification accuracy when labelled data is limited. Additionally, VAEs support semi-supervised learning, enabling them to utilize both labelled and unlabeled data, which is a significant advantage over purely supervised models. Their generative nature also enables data augmentation through the generation of synthetic samples, thereby addressing class imbalance issues. Overall, VAEs provide a flexible, robust, and interpretable framework that often outperforms standard classifiers in complex, real-world scenarios.

VAE is employed for learning a latent compressed representation of transactions. Anomalies are recognized to reconstruct transactions and measure reconstruction errors. The method maps transaction information into a latent space and then maps it back, thereby minimizing the loss of reconstruction while preserving significant transaction patterns.

The encoder network projects input transactions into a latent space representation over deep neural layers. It generates a probabilistic distribution to assess the variance and mean of latent embeddings. A reparameterization trick is employed to sum stochastic units, safeguarding robust anomaly recognition.

The encoder converts transaction data $\:\mathcal{X}$ into a latent representation $\:z$:

13 $$\:z\:=\:{\mu\:}_{\theta\:}+{\sigma\:}_{\theta\:}\cdot\:\:\eta\:,\:\eta\:\:\sim\:\:\mathcal{N}\left(0,I\right)$$

Here, $\:\eta\:$ represents an arbitrary noise sampled from a standard normal distribution, $\:{\mu\:}_{\theta\:}$, and $\:{\sigma\:}_{\theta\:}$ refer to learned latent space parameters.

The decoder constructs transactions from the latent representations generated by the encoder. It reduces the discrepancy between the original and reconstructed transactions due to the loss of reconstruction. While the reconstruction error for a transaction is higher, it is subsequently viewed as a likely fraud candidate, depending on its typicality compared to standard transaction profiles. The decoder rebuilds the original transaction information:

14 $$\:\widehat{\mathcal{X}}\:=\:{g}_{\varphi\:}\left(z\right)$$

Now $\:\widehat{\mathcal{X}}$ depicts the reconstructed output.

The VAE loss function is separated into dual parts: Reconstruction Loss that confirms the reconstructed transaction is similar to the original, and Kullback–Leibler (KL) Divergence that is employed to regularise the latent space distribution. This loss function aids the method to learn better discrimination between anomalous and normal transactions.

The VAE decreases the integrated KL divergence and reconstruction loss:

15 $${\mathcal{L}}_{{VAE}} = {\mathbb{E}}_{{q\left( {z{\mathcal{X}}} \right)}} ~\left[ {log~p\left( {{\mathcal{X}\mid ~}z} \right)} \right] - {\mathbb{D}}_{{KL}} \left( {q(z\;{\mathcal{X}})p(z)} \right)$$

Here, $\:{\mathbb{D}}_{KL}$ determines the divergence from the normal prior.

## Experimental analysis

**Fig. 3:**
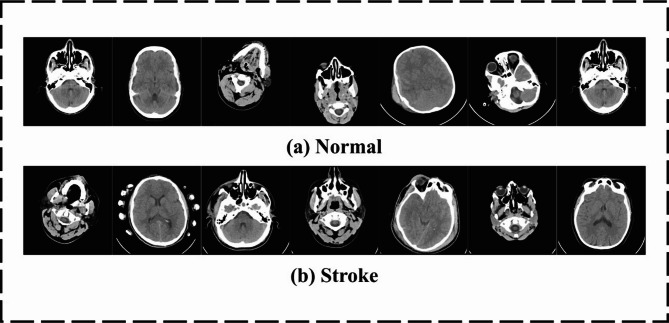
Sample images (**a**) normal and (**b**) stroke.

**Fig. 4:**
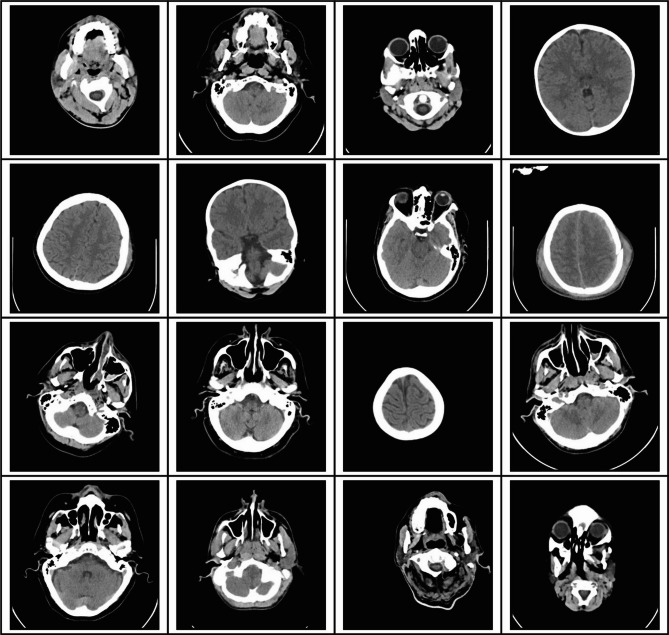
Sample images.

In this section, the EBSDC-AIFFT technique is examined under the brain stroke CT image dataset^42^. The extracted features include lesion texture, intensity shifts, edge sharpness, and shape irregularities. Inception-ResNet-v2 captures multi-scale spatial and boundary features, while CBAM-ResNet18 improves stroke-relevant regions through channel and spatial attention. MaxViT encodes global context and positional dependencies, and the VAE compresses these into latent vectors preserving asymmetry and abnormal tissue patterns significant for stroke classification. The method runs on Python 3.6.5 with an i5-8600k CPU, 4GB GPU, 16GB RAM, 250GB SSD, and 1 TB HDD, using a 0.01 learning rate, ReLU, 50 epochs, 0.5 dropout, and batch size 5. Table 2 indicates the dataset description. Figure 3 shows sample images of normal and stroke conditions. Figure 4 depicts the sample images.

**Table 2:** Dataset details.

Classes	CT Images
“Normal”	“1551”
“Stroke”	“950”
Total	2501

**Fig. 5:**
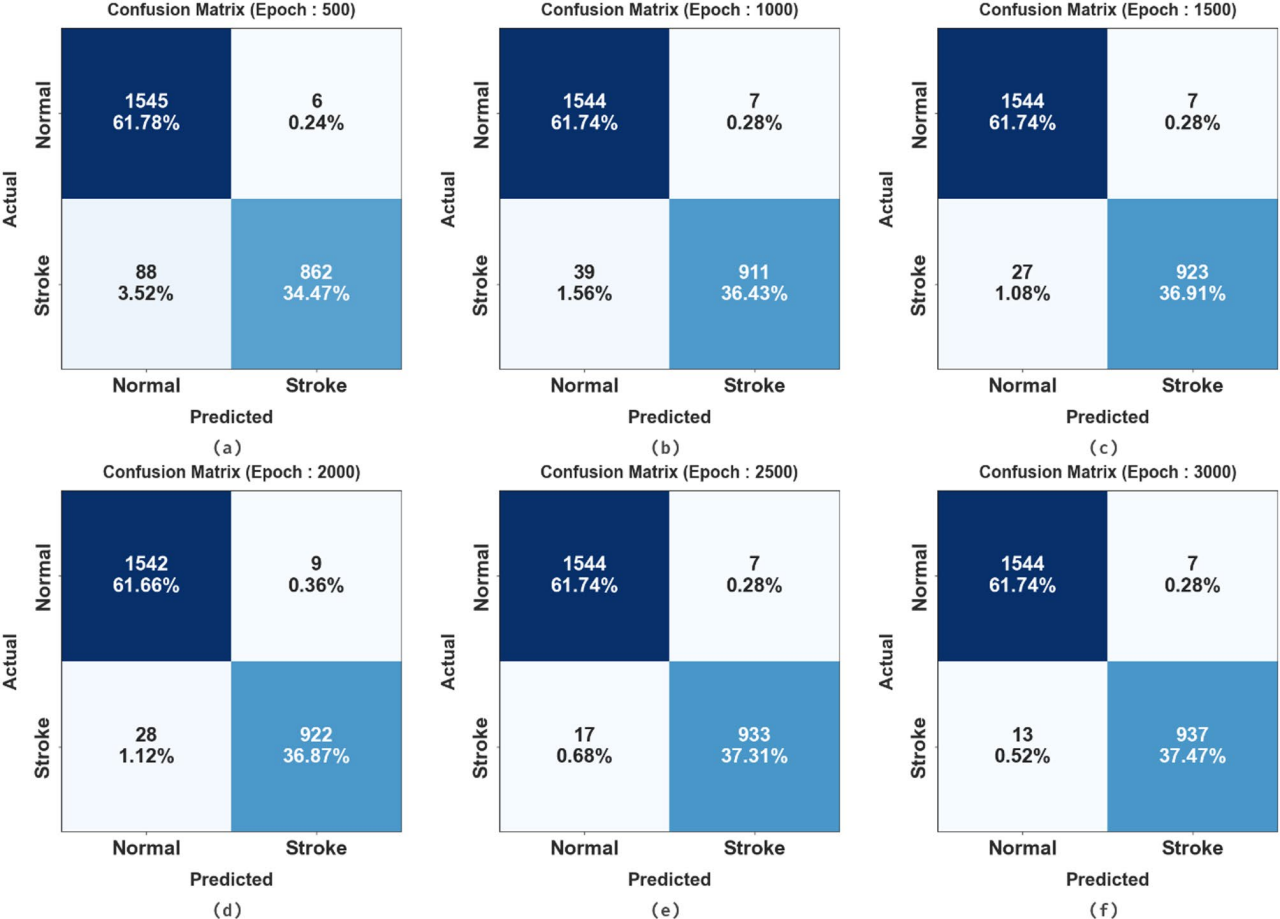
Confusion matrices of EBSDC-AIFFT model (a-f), Epochs 500 to 3000.

Figure 5 illustrates the confusion matrices generated by the EBSDC-AIFFT method at various epoch counts. The outcomes denote that the EBSDC-AIFFT approach effectively identifies and recognizes each class.

**Fig. 6:**
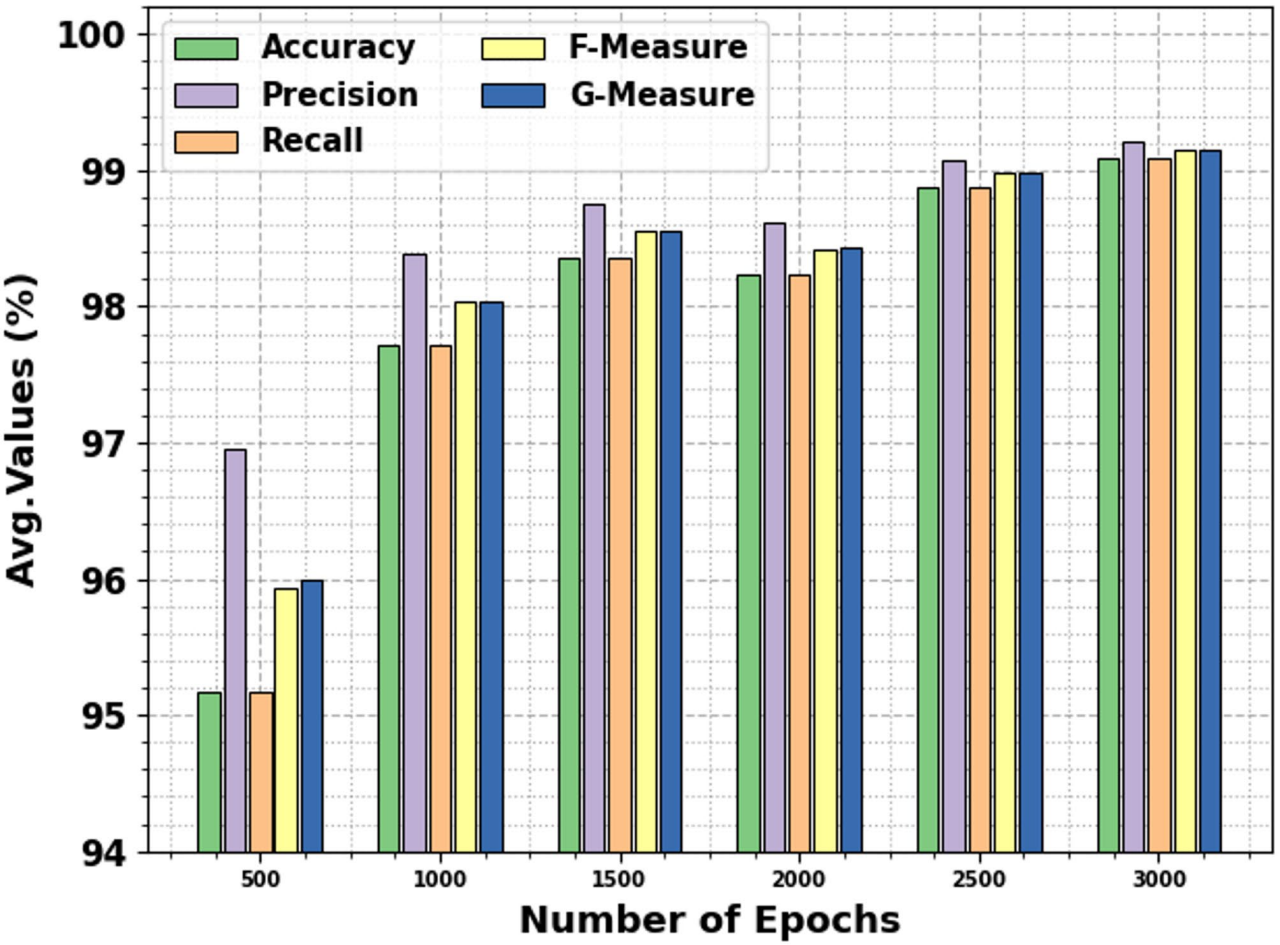
Average values of EBSDC-AIFFT (a-f), Epochs 500–3000.

The brain stroke detection of the EBSDC-AIFFT technique is established on different epochs in Table 3; Fig. 6. The table values indicate that the EBSDC-AIFFT technique correctly recognized every sample. Under 3000 epochs, the EBSDC-AIFFT model presents superior average $\:acc{u}_{y}$ of 99.09%, $\:pre{c}_{n}\:$of 99.21%, $\:rec{a}_{l}$ of 99.09%, $\:{F}_{Measure}$ of 99.15%, and $\:{G}_{Measure}$of 99.15%.

**Table 3:** Brain stroke detection of the EBSDC-AIFFT model under diverse epochs.

Class Labels	$\:\varvec{A}\varvec{c}\varvec{c}{\varvec{u}}_{\varvec{y}}$	$\:\varvec{P}\varvec{r}\varvec{e}{\varvec{c}}_{\varvec{n}}$	$\:\varvec{R}\varvec{e}\varvec{c}{\varvec{a}}_{\varvec{l}}$	$\:{\varvec{F}}_{\varvec{M}\varvec{e}\varvec{a}\varvec{s}\varvec{u}\varvec{r}\varvec{e}}$	$\:{\varvec{G}}_{\varvec{M}\varvec{e}\varvec{a}\varvec{s}\varvec{u}\varvec{r}\varvec{e}}$
Epoch − 500					
Normal	99.61	94.61	99.61	97.05	97.08
Stroke	90.74	99.31	90.74	94.83	94.93
Average	95.17	96.96	95.17	95.94	96.00
Epoch − 1000					
Normal	99.55	97.54	99.55	98.53	98.54
Stroke	95.89	99.24	95.89	97.54	97.55
Average	97.72	98.39	97.72	98.03	98.04
Epoch − 1500					
Normal	99.55	98.28	99.55	98.91	98.91
Stroke	97.16	99.25	97.16	98.19	98.20
Average	98.35	98.76	98.35	98.55	98.56
Epoch − 2000					
Normal	99.42	98.22	99.42	98.81	98.82
Stroke	97.05	99.03	97.05	98.03	98.04
Average	98.24	98.62	98.24	98.42	98.43
Epoch − 2500					
Normal	99.55	98.91	99.55	99.23	99.23
Stroke	98.21	99.26	98.21	98.73	98.73
Average	98.88	99.08	98.88	98.98	98.98
Epoch − 3000					
Normal	99.55	99.17	99.55	99.36	99.36
Stroke	98.63	99.26	98.63	98.94	98.94
Average	99.09	99.21	99.09	99.15	99.15

**Fig. 7:**
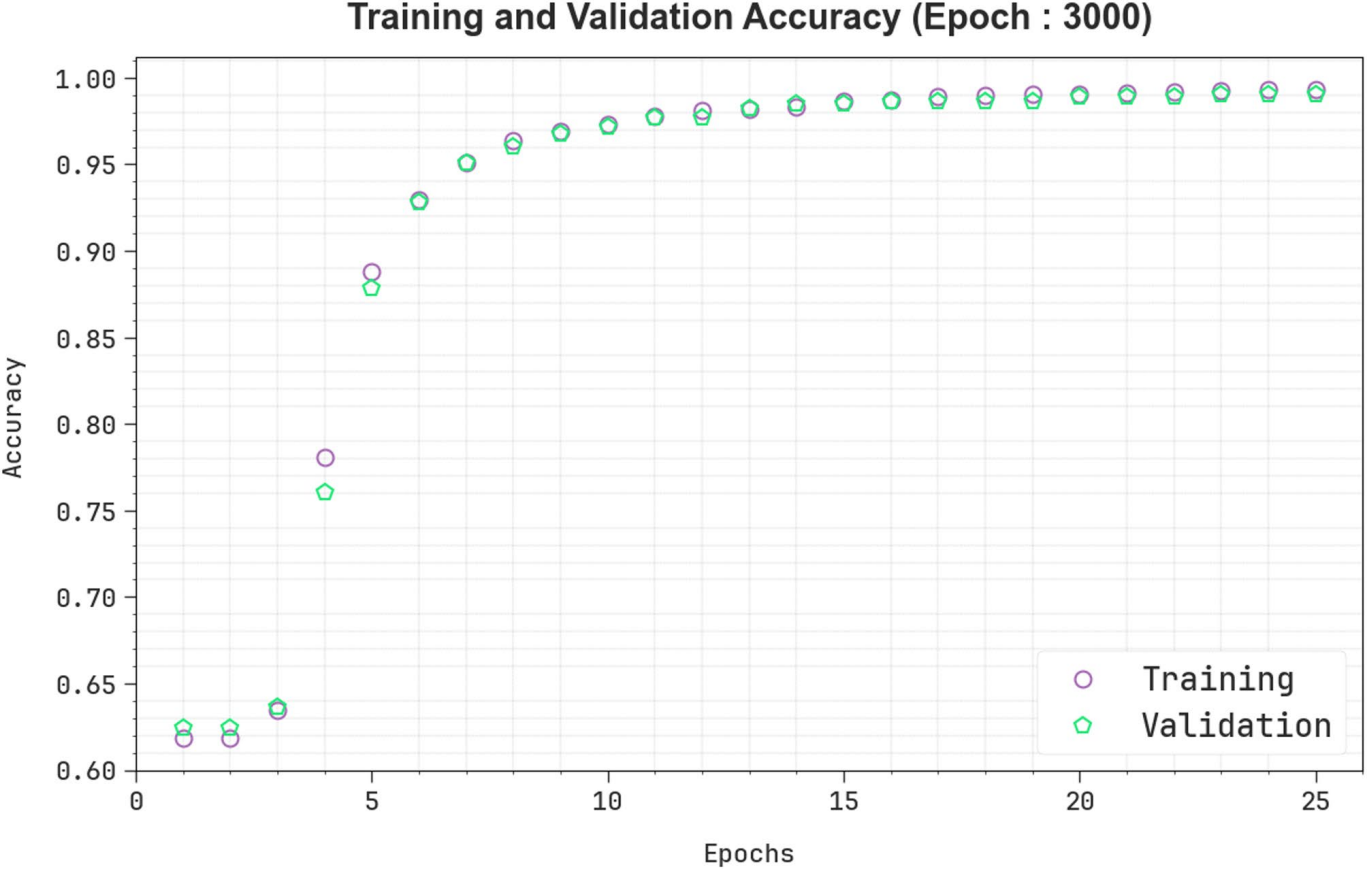
$\:Acc{u}_{y}$ curve of the EBSDC-AIFFT method under Epoch 3000.

Figure 7 demonstrates the training (TRAIN) $\:acc{u}_{y}$ and validation (VALID) $\:acc{u}_{y}$ of an EBSDC-AIFFT technique under Epoch 3000. At first, both TRAIN and VALID $\:acc{u}_{y}\:$rise quickly, specifying efficient pattern learning from the data. Around the epoch, the VALID $\:acc{u}_{y}$ slightly exceeds the training accuracy, signifying good generalization without overfitting. As training evolves, it reflects a narrower performance gap between TRAIN and VALID. The close alignment of both curves across training indicates that the model is well-regularised and well-generalized. This establishes the model’s robust capability to learn and retain beneficial features among both seen and unseen data.

**Fig. 8:**
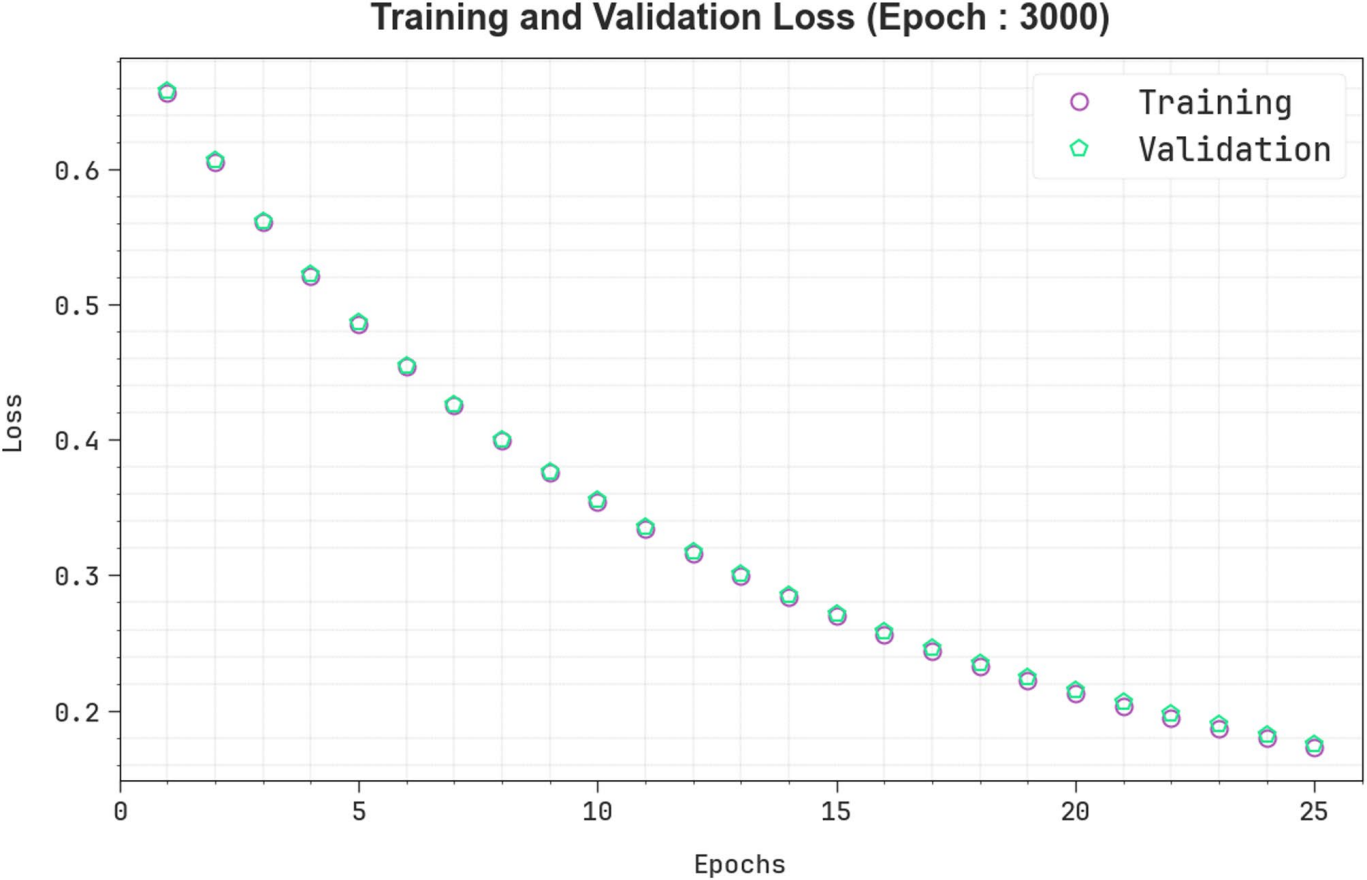
Loss curve of EBSDC-AIFFT model under Epoch 3000.

Figure 8 exemplifies the TRAIN and VALID losses of the EBSDC-AIFFT approach under Epoch 3000. Initially, both TRAIN and VALID losses are higher, demonstrating that the model begins with a limited understanding of the data. As training evolves, both losses persistently decrease, indicating that the model is efficiently learning and refining its parameters. The close alignment between the TRAIN and VALID loss curves throughout training suggests that the model hasn’t overfitted and retains good generalization to unseen data. This reliable and steady decrease in loss shows a stable, well-trained, and consistent deep-learning model.

**Fig. 9:**
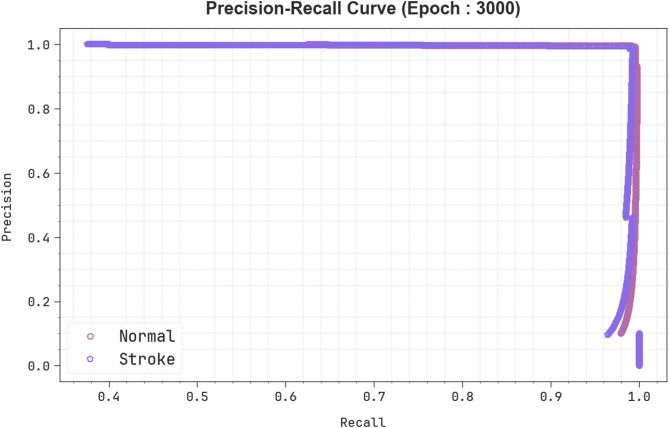
PR curve of EBSDC-AIFFT model under Epoch 3000.

In Fig. 9, the precision-recall (PR) inspection analysis of the EBSDC-AIFFT method under Epoch 3000 provides insight into its performance through charting PR for every label. The figure shows that the EBSDC-AIFFT technique maintains high PR across classes, highlighting its effective and consistent classification performance.

**Fig. 10:**
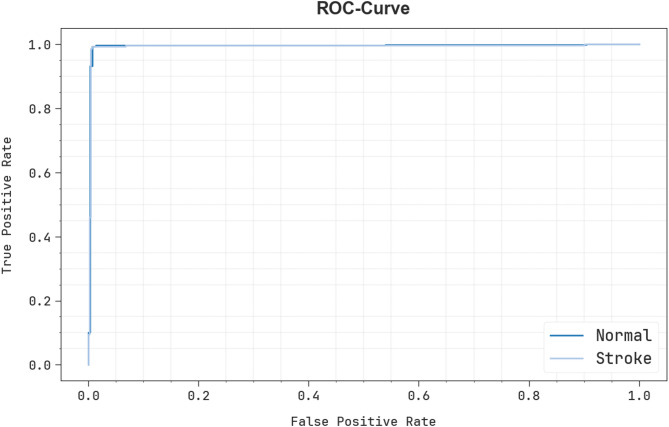
ROC curve of EBSDC-AIFFT model under Epoch 3000.

In Fig. 10, the ROC of the EBSDC-AIFFT technique under Epoch 3000 is examined. The findings indicate that the EBSDC-AIFFT technique achieves superior ROC outcomes across all classes, demonstrating its ability to differentiate between classes effectively. This steady pattern of increased ROC values across several classes indicates the efficacious outcomes of the EBSDC-AIFFT model in class prediction, underscoring the efficiency.

**Fig. 11:**
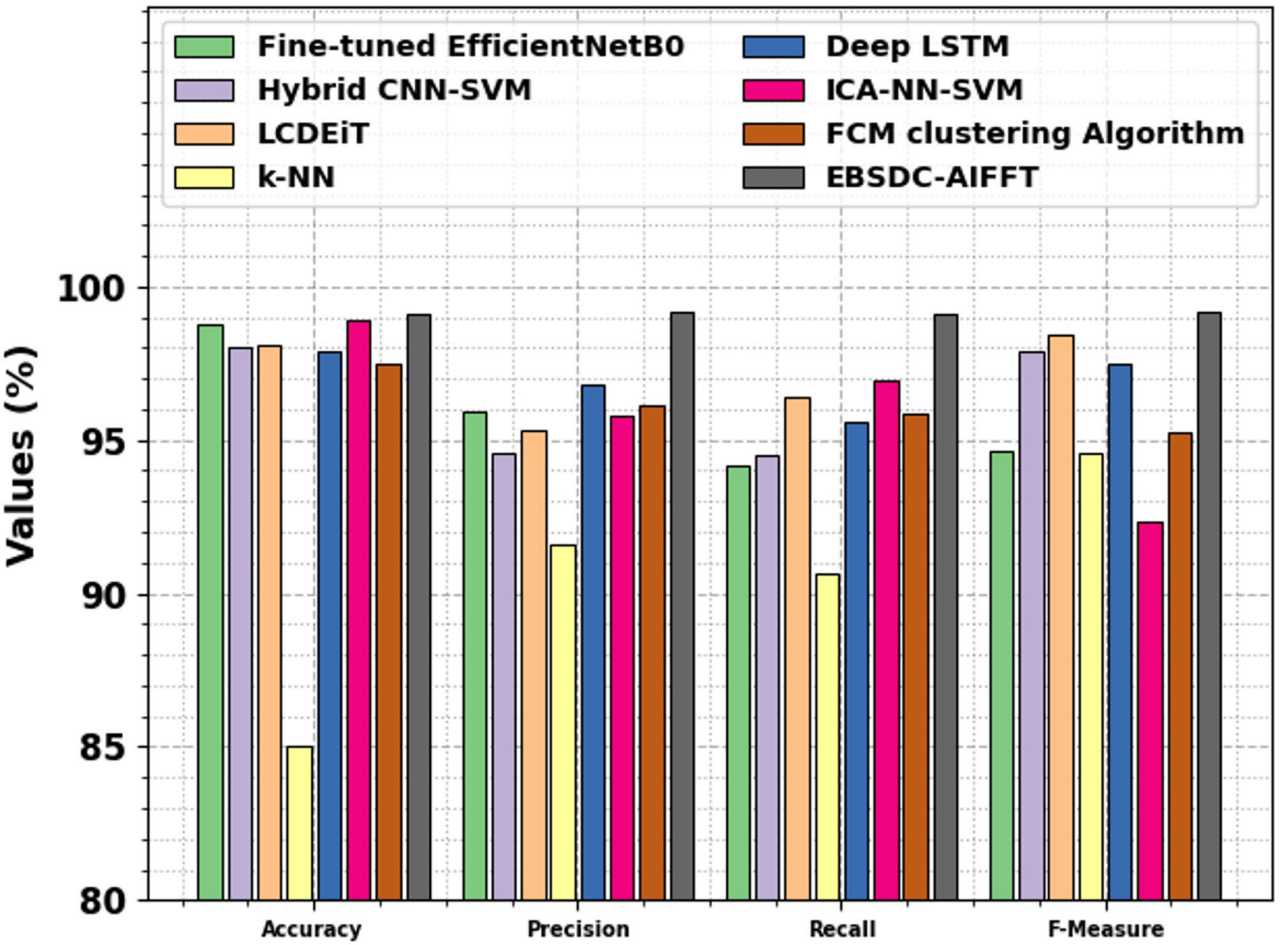
Comparative study of EBSDC-AIFFT model with existing approaches.

The comparative analysis of the EBSDC-AIFFT method with recent models is portrayed in Table 4; Fig. 11^43,44^. The simulation outcome specified that the EBSDC-AIFFT method outperformed other models. On $\:acc{u}_{y}$, the EBSDC-AIFFT method attains the highest $\:acc{u}_{y}$ of 99.09%. In contrast, the Fine-tuned EfficientNetB0, Hybrid CNN-SVM, LCDEiT, k-NN, Deep LSTM, ICA-NN-SVM, and FCM clustering methodologies achieved a lower $\:acc{u}_{y}$ of 98.80%, 98.01%, 98.11%, 85.00%, 97.87%, 98.90%, and 97.50%, correspondingly. Similarly, based on $\:pre{c}_{n}$, the EBSDC-AIFFT model has obtained a higher $\:pre{c}_{n}$ of 99.21% while the Fine-tuned EfficientNetB0, Hybrid CNN-SVM, LCDEiT, k-NN, Deep LSTM, ICA-NN-SVM, and FCM clustering methodologies have obtained a lower $\:pre{c}_{n}$ of 95.95%, 94.57%, 95.32%, 91.58%, 96.83%, 95.79%, and 96.14%, respectively. Finally, based on $\:{F}_{Measure}$, the EBSDC-AIFFT model gained the highest $\:{F}_{Measure}$ of 99.15%. In contrast, the Fine-tuned EfficientNetB0, Hybrid CNN-SVM, LCDEiT, k-NN, Deep LSTM, ICA-NN-SVM, and FCM clustering methodologies have a lower $\:{F}_{Measure}$ of 94.65%, 97.90%, 98.45%, 94.58%, 97.48%, 92.37%, and 95.22%, respectively.

**Table 4:** Comparative study of EBSDC-AIFFT model with existing approaches.

Method	$\:\varvec{A}\varvec{c}\varvec{c}{\varvec{u}}_{\varvec{y}}$	$\:\varvec{P}\varvec{r}\varvec{e}{\varvec{c}}_{\varvec{n}}$	$\:\varvec{R}\varvec{e}\varvec{c}{\varvec{a}}_{\varvec{l}}$	$\:{\varvec{F}}_{\varvec{M}\varvec{e}\varvec{a}\varvec{s}\varvec{u}\varvec{r}\varvec{e}}$
Fine-tuned EfficientNetB0	98.80	95.95	94.15	94.65
Hybrid CNN-SVM	98.01	94.57	94.54	97.90
LCDEiT	98.11	95.32	96.37	98.45
k-NN	85.00	91.58	90.62	94.58
Deep LSTM	97.87	96.83	95.58	97.48
ICA-NN-SVM	98.90	95.79	96.93	92.37
FCM clustering algorithm	97.50	96.14	95.88	95.22
EBSDC-AIFFT	99.09	99.21	99.09	99.15

**Fig. 12:**
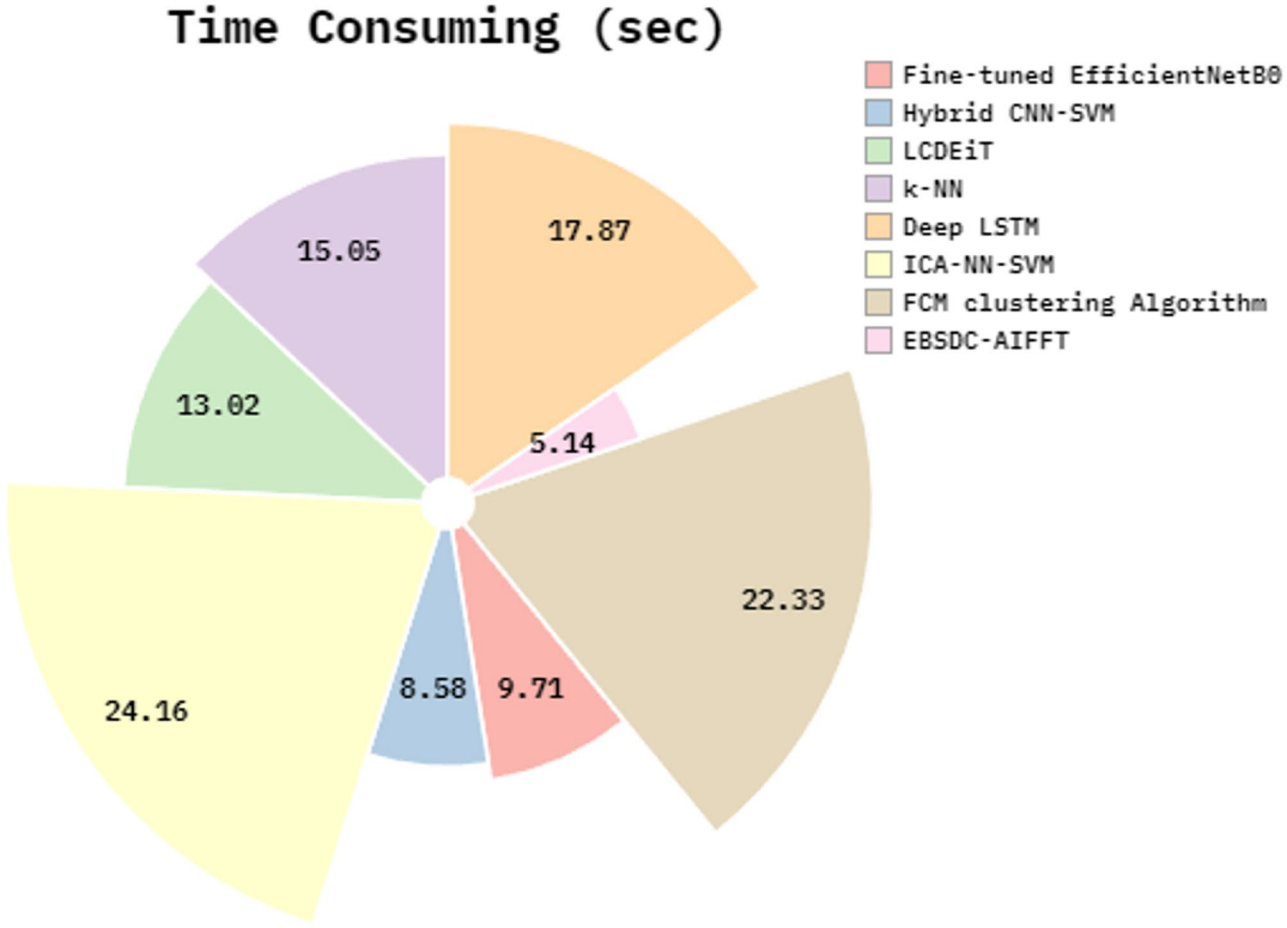
TC outcome of EBSDC-AIFFT model with existing methods.

The time consumption (TC) of the EBSDC-AIFFT approach with current models is demonstrated in Table 5; Fig. 12. The EBSDC-AIFFT technique achieves a lower TC of 5.14 min. In contrast, the Fine-tuned EfficientNetB0, Hybrid CNN-SVM, LCDEiT, k-NN, Deep LSTM, ICA-NN-SVM, and FCM clustering approaches got superior TC of 9.71 min, 8.58 min, 13.02 min, 15.05 min, 17.87 min, 24.16 min, and 22.33 min, respectively.

**Table 5:** TC outcome of EBSDC-AIFFT model with existing methods.

Method	TC (min)
Fine-tuned EfficientNetB0	9.71
Hybrid CNN-SVM	8.58
LCDEiT	13.02
k-NN	15.05
Deep LSTM	17.87
ICA-NN-SVM	24.16
FCM Clustering Algorithm	22.33
EBSDC-AIFFT	5.14

## Conclusion

This paper develops an EBSDC-AIFFT model. The paper aims to create an enhanced brain stroke detection system for individuals with disabilities, utilizing biomedical images to improve diagnostic accuracy. Initially, the image pre-processing stage involves several steps, including data augmentation, data splitting, normalization, and resizing, to enhance image quality. Additionally, the EBSDC-AIFFT model integrates the Inception-ResNet-v2 model, the CBAM-ResNet18 method, and the MaxViT method for feature extraction. Finally, the VAE technique is applied to the classification procedure. The performance validation of the EBSDC-AIFFT technique is performed under the brain stroke CT image dataset. The comparison study of the EBSDC-AIFFT technique demonstrated a superior accuracy value of 99.09% over existing models. The limitations of the EBSDC-AIFFT technique comprise the reliance on a single imaging modality, which may restrict generalizability across varied clinical scenarios. The dataset size, although sufficient for preliminary evaluation, may not capture rare stroke variants or demographic diversity. The performance may vary in real-world clinical settings due to limited external validation. The absence of multi-center data and integration with electronic health records restricts its broader applicability. Future work may concentrate on incorporating multimodal data, expanding datasets with more heterogeneous samples, and improving interpretability for clinical acceptance. Real-time deployment in clinical environments will be explored. Efforts will also be directed toward regulatory compliance and ethical considerations.

## Data Availability

The data that support the findings of this study are openly available in the Kaggle repository at https://www.kaggle.com/datasets/afridirahman/brain-stroke-ct-image-dataset, reference number^42^.
